# Infertile men older than 40 years are at higher risk of sperm DNA damage

**DOI:** 10.1186/1477-7827-12-103

**Published:** 2014-11-20

**Authors:** Saad Alshahrani, Ashok Agarwal, Mourad Assidi, Adel M Abuzenadah, Damayanthi Durairajanayagam, Ahmet Ayaz, Rakesh Sharma, Edmund Sabanegh

**Affiliations:** Glickman Urological and Kidney Institute, Center for Reproductive Medicine, Cleveland Clinic, Cleveland, OH USA; College of Medicine, Salman Bin Abdulaziz University, Alkharj, Saudi Arabia; College of Medicine, Salman Bin Abdulaziz University, Jeddah, Saudi Arabia; Center of Excellence in Genomic Medicine Research, King AbdulAziz University, Jeddah, Saudi Arabia; KACST Technology Innovation Center in Personalized Medicine at King AbdulAziz University, Jeddah, Saudi Arabia; MARA University of Technology, Selangor Darul Ehsan, Malaysia

**Keywords:** Paternal age, Sperm DNA damage, Male infertility, Semen parameters

## Abstract

**Background:**

The effect of paternal age on semen quality is controversial. In this retrospective study, the aim was to investigate the effects of advancing age on sperm parameters including reactive oxygen species (ROS), total antioxidant capacity (TAC) and sperm DNA damage in infertile men. We also examined whether paternal age >40 y is associated with higher risk of sperm DNA damage.

**Methods:**

A total of 472 infertile men presenting for infertility were divided into 4 age groups: group A: patients ≤ 30 y; group B: patients 31- 40 y, group C: ≤ 40 y and group D: patients >40 y. The following tests were performed - semen analysis according to WHO 2010 criteria, seminal ROS by chemiluminescence, TAC by colorimetric assay and sperm DNA damage by TUNEL assay - and the results were compared amongst the 4 age groups.

**Results:**

There was no statistical difference in conventional semen parameters, TAC and ROS with advancing paternal age as well as between different age groups. However, a significant negative association was noted between sperm DNA damage and advancing paternal age. Men >40 y showed higher levels of sperm DNA damage (24.4 ± 18.5%) compared to younger men (<30 y; 16.7 ± 11.2%; p <0.05).

**Conclusions:**

Infertile men over the age of 40 y have a greater percentage of sperm DNA fragmentation compared to infertile men aged 40 y and below. Advanced paternal age (>40 y) may increase the risk of sperm DNA damage in infertile men.

## Background

Many couples in developed countries are delaying parenthood for a variety of reasons [1–3]. Most believe that delayed parenthood has many advantages [4]. In England and Wales, 25% of live births in 1993 were to fathers older than 35 y but after 10 years, the percentage increased to 40% [5]. In the USA, birth rates for men older than 35 y have increased 40% since 1980 [2, 6].

The effect of maternal ageing on fertilization and reproduction is well known [7]. Several studies have shown that women over 35 y have a higher risk of infertility, pregnancy complications, spontaneous abortion, congenital anomalies, and perinatal complications [2, 8–10]. On the other hand, the effect of paternal age on semen quality is controversial for a couple of reasons. First, there is no universal definition for advanced paternal ageing. The mean population age for paternal age is 21 y, and 40 y is the most frequently used cutoff to describe advanced paternal ageing [2].

Secondly, the literature is full of studies with conflicting results, especially for the most common parameters tested (volume, concentration, motility, total sperm count, morphology) [11–17]. A recent meta-analysis showed a consistent impact of advanced age on semen volume but the effect on the other semen parameters was inconsistent [18]. Advancing paternal age has a negative impact on semen volume [15, 19, 20], sperm motility [19, 20], and normal morphology [19, 21, 22]. Sperm concentration did not show any correlation with male age [23, 24] while another report showed an increase with age [25].

Advancing paternal age also has been associated with increased risk of genetic disease [26, 27], schizophrenia [28], autism [26], and other complex disorders [29]. Several studies show that advanced paternal age increases the risk of spontaneous abortions [30, 31], and increased risk of low birth weight [32]. De La Rochebrochard and Thonneau found that men who were older than 40 y were at high risk for infertility [33]. The same group also reported higher risk of infecundity and miscarriages in women ≥35 y and men ≥40 y [34].

One possible explanation for the negative effects of advanced paternal age on reproductive outcome is sperm DNA damage [21, 35]. Intact sperm DNA is essential for fertilization and for the genetic transmission to the next generation [23, 36]. Sperm DNA damage is associated with reduced fertility [37], increased miscarriage rates [38], abnormal embryonic development [39], and compromised chromosomal integrity in the embryo [40]. Even though the effect of advanced paternal age on sperm DNA damage has been studied, the results are inconsistent as different age groups and different measurement techniques were used [23, 24, 41–43].

The aims of this study were to assess the effects of advancing age on sperm parameters including reactive oxygen species, total antioxidant capacity and sperm DNA damage in infertile men and investigate if a paternal age of >40 y is associated with a higher risk of sperm DNA damage.

## Methods

### Subjects

We conducted a retrospective review of the medical records of patients who presented to our Andrology clinic with a history of infertility of at least 1 y. The Cleveland Clinic Institutional Review Board had approved this study. We examined the medical records of patients attending the Andrology laboratory for semen analysis between 2010 to September 2012. The purpose of this study was to explore the overall effect of ageing in non-azoospermic infertile men (n = 472) regardless of their type of infertility. Patients were assigned to 4 groups based on their age: group A: patients ≤30 y (n = 69; 14.6%), group B: patients 31-40 y (n = 298; 63.2%), C: ≤40 y and group D: patients >40 y (n = 105; 22.2%). All patients underwent a detailed medical history and physical examination. Incidence and duration of primary and secondary infertility was recorded. Primary infertility is defined when no pregnancy is established at any point by the couple. Secondary infertility is when a biological pregnancy has been established once but subsequent pregnancies cannot be established in the same couple.

Conventional semen parameters (semen volume, concentration, motility, and normal morphology), total antioxidant capacity (TAC), seminal reactive oxygen species (ROS), and sperm DNA damage were noted. We also collected additional information regarding the presence/stage of varicocele, alcohol and cigarette use. All partners of these men were ruled out for any female-factor infertility. Semen samples were obtained from patients who were identified with varicocele and had not undergone surgery for varicocele repair.

### Semen analysis

Semen samples were collected by masturbation after 3-5 days of sexual abstinence. Five μL of a liquefied sample was loaded on a 20 micron MicroCell slide (Vitrolife, San Diego, CA). A minimum of 200 spermatozoa were examined in each sample. The conventional semen parameters such as sperm concentration, percentage motility and normal sperm morphology were assessed according to the 2010 World Health Organization 5^th^ edition criteria [44].

### Total antioxidant capacity (TAC)

Following completion of initial semen analysis, an aliquot of the sample was centrifuged at 1600 rpm for 7 minutes. Clear supernatant was removed and batched and stored at – 80°C for measurement of seminal antioxidant concentrations. The total antioxidant capacity (TAC) of the seminal plasma was measured using an antioxidant assay kit (Cayman Chemical Company, Ann Arbor, MI). Its principle is based on the ability of aqueous- and lipid-based antioxidants in seminal plasma to inhibit oxidation of the ABTS (2,2′-Azino-di-[3-ethylbenzthiazoline sulphonate]) to ABTS^•+^. Frozen seminal plasma samples and the contents of the assay kit were removed and thawed at room temperature (25°C) for 20 minutes. Seminal plasma vials were centrifuged at 5,000 rpm for 7 minutes. Clear seminal plasma was removed into labeled 2 mL eppendorf tubes. The seminal plasma was diluted with assay buffer (1:10 vol./vol.). For the assay, 10 μL of the diluted seminal plasma and the standard (trolox) were run in duplicate. 10 μL of metmyoglobin and 150 μL of chromogen were added to each well. The reaction was initiated by adding of 40 μL of hydrogen peroxide (working solution) using a multi-channel pipette as described earlier [45]. The antioxidants present in the seminal plasma suppress absorbance to a degree that is proportional to their concentration. The measurement was read at 750 nm using Microplate Reader (Epoch BioTek Gen 5 Absorbance; BioTek Instruments, Inc., Winooski, VT). The results were expressed as micromolar trolox equivalents [45].

### Reactive oxygen species (ROS)

Seminal ROS was measured in the liquefied seminal ejaculates without any further processing with a chemiluminescence assay. Luminol (5 mM; 5-amino-2, 3-dihydro-1, 4-phthalazinedione; Sigma Chemical Co., St. Louis, MO) was used as the probe. Test samples consisted of luminol (10 μL, 5 mM) and 400 μL of liquefied seminal ejaculate. Negative controls were prepared by replacing the sperm sample with 400 μL phosphate buffered saline. Positive control included 400 μL of PBS and 50 μL of hydrogen peroxide (30%; 8.8 M) in triplicate. Chemiluminescence was measured for 15 min using a Berthold luminometer (Autolumat Plus 953; Oakridge, TN). Results were expressed as relative light units (RLU)/sec. Sperm concentration was calculated and final ROS levels were adjusted by dividing with the sperm concentration to represent the final levels of ROS (RLU/sX10^6^ sperm [46].

### Sperm DNA damage

Sperm DNA damage was evaluated by the terminal deoxynucleotidyl transferase-mediated deoxyuridine biotin nick-end labeling (TUNEL) assay with an Apo-Direct kit (Pharmingen, San Diego, CA). The sperm concentration was adjusted to 5 × 10^6^ sperm/mL. The TUNEL assay involves multiple washing and resuspending steps and some sperm are lost at each step. Spermatozoa were washed in phosphate-buffered saline (PBS) and the spermatozoa were re-suspended in ice-cold 3.7% paraformaldehyde for 30-60 minutes. Paraformaldehyde was removed by centrifugation and re-suspended in 70% ice-cold ethanol. Before staining, sperm were washed twice in ‘Wash buffer’ and re-suspended in staining solution (50 μL for 60 minutes at 37°C) as per manufacturer’s instructions. After incubation, samples were washed twice with 1 mL of ‘Rinse buffer’. 0.5 mL of propidium iodine/RNase solution was added and samples were incubated for 30 minutes before the flow cytometric analysis to measure positive cells for DNA damage [47]. Both positive and negative controls were included with each run. Positive controls consisted of spermatozoa treated with DNase I. Negative controls were prepared by omitting TdT from the staining solution. The results were expressed as the percentage of sperm with DNA damage.

Spermatozoa/events obtained in the plots were gated using a forward-angle light scatter (FSC) and a side-angle light scatter (SSC) dot plot to gate out debris, aggregates, and other cells different from spermatozoa. TUNEL-positive spermatozoa in the population were measured after converting the data into a histogram. The percentage of positive cells (TUNEL-positive) were calculated on a 1,023-channel scale using the flow cytometer software FlowJo Mac version 8.2.4 (FlowJo, LLC, Ashland, OR) [47].

### Statistical analysis

Fisher’s exact test or Chi square was used to compare groups of qualitative variables (infertility- primary or secondary). The Kruskal-Wallis test was used to compare groups of quantitative variables (abstinence, volume, concentration, motility, and morphology, duration of infertility, ROS, TAC and DNA damage). Data are represented as mean ± standard deviation (SD) for all the variables except ROS, which was represented as median (25^th^; 75^th^ percentile). A *P* value <0.05 was considered statistically significant.

## Results

The study group was composed of 472 non-azoospermic infertile men who presented to our andrology clinic. All patients were advised of 2-3 days of abstinence before providing a semen sample. In this study the overall abstinence time was 3.8 ± 2.0 days. It was 3.8 ± 2.8 days in the <30 y; 3.7 ± 1.5 days in 31-40y and 4.2 ± 2.6 days in >40 y group. The age ± standard deviation (SD) was 36.8 ± 6.7 y. The mean, median, and range of ages in the 4 groups was: ≤30 y: mean = 28.2 y, median = 29 y, range = (22 y, 30 y); 31 - 40 y: mean = 35.3 y, median = 35 y, range = (31 y, 40 y) and >40 y: mean = 46.6 y, median = 45 y, range = (41 y, 68 y). The overall infertility duration of the men in our study was 1.2 ± 0.6 y; 1.1 ± 0.4 y in ≤30 y group; 1.2 ± 0.6 y in 31-40 y group; 1.2 ± 0.5 y in <40 y and 1.4 ± 0.7 y in >40 y group. A significant difference was noted in the duration of infertility between <30 y vs. >40 y group (p = 0.004) and 31 - 40 y vs. >40y (p <0.012).

375 of 471 of the patients (79.6%) presented with primary infertility while only 96 of 471 (20.4%) presented with secondary infertility. Duration of primary infertility in our study was higher in men >40 y compared to those ≤30 y. The overall duration of infertility (primary and secondary) was 2.3 ± 1.9 y. In the majority of these patients 192/460 (41.7%) the duration of infertility was 1 y, while in 150/460 (32.6%) the duration was 2 y. Overall, 84.6% of the patients (389/460) had infertility duration of 1-3 y. Of the patients, 77.8% (367/472) were ≤40 y and 22.2% (105/472) were >40 y. Significant differences were seen between the duration of infertility and the different age groups. 92.5% (62/67) of men ≤30 y had 1-3 y of infertility, while this number significantly decreased to 74.7% (74/99) in men >40 y. Inversely the duration of infertility >5 y increased from 3% in men ≤30 y to 11.1% in men >40 y.

**Figure 1:**
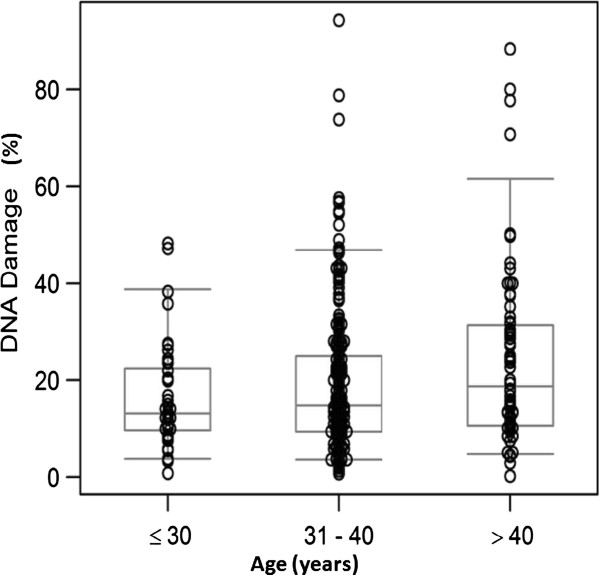
**DNA damage in the different age groups of infertile men.** Percentage of DNA damage assessed using the TUNEL method is shown on the Y-axis and age group on the X-axis. Significantly higher levels of DNA damage was seen in >40 y compared to the ≤30 y.

The overall mean ± standard deviation (SD) for various sperm parameters in the 4 age groups is shown in Table 1. No significant differences were seen in the conventional semen parameters, TAC and ROS levels in the 4 age groups. However, a significant increase in sperm DNA damage was seen with advancing paternal age (Figure 1). Sperm DNA damage was statistically significantly higher in patients >40 y compared to the younger patients. When the patients were grouped into 2 groups i.e. ≤40 y and >40 y, semen parameters were comparable between the overall as well as the two groups (Table 1). However, higher levels of DNA damage were seen in men >40 y when compared with men ≤40 y (P < 0.05) as well as in the overall group (P < 0.01).

**Table 1:** **Comparison of semen parameters between overall and 4 age groups**

Parameters	Overall	≤30 y	31 – 40 y	<40 y	>40 y
		(n = 69)	(n = 298)	(n = 367)	(n = 105)
	(n = 472)	69/472 (14.6%)	298/472 (63.1%)	367/472 (77.8%)	105/472 (22.2%)
Volume (mL)	3.1 ± 1.5	3.4 ± 1.5	3 ± 1.4	3.1 ± 1.4	3.1 ± 1.75
Concentration (X10^6^/mL)	42.01 ± 50.58	36.66 ± 39.72	42.62 ± 52.23	41.50 ± 50.12	43.80 ± 52.37
Motility (%)	44.7 ± 19.7	44.3 ± 14.4	45.2 ± 19.5	45.0 ± 18.6	43.5 ± 22.9
Normal Morphology (%)	3.2 ± 2.9	3.1 ± 2.7	3.1 ± 2.9	3.1 ± 2.9	3.3 ± 3.1
TAC (micromolar trolox)	1964.42 ± 683.77	2114.02 ± 548.93	1948.80 ± 689.78	1974.03 ± 671.09	1930.87 ± 727.40
ROS (RLU/sec/X10^6^)	267.7 (59.3; 1277.3)	311.1 (38.4; 1927.3)	256.9 (65.9; 1149.1)	265.6 (63.8; 1204.1)	429.9 (54.9; 1514.0)
Sperm DNA damage (%)	19.9 ± 15.3	16.7 ± 11.2	19.1 ± 14.6	18.7 ± 14. 1	24.4 ± 18.5^a,b,c,d^

The results are presented as mean ± SD for all the parameters except ROS which is presented as median (25^th^; 75^th^ percentile).

^a^ *P* value <0.05 when >40 y group was compared with the overall group.

^b^ *P* value <0.05 when >40 y group was compared with the group ≤30 y.

^c^ *P* value <0.05 when >40 y group was compared with the group 31-40 y

^d^ *P* value <0.05 when >40 y group was compared with the group <40 y.

Only 21.7% (100/460) of the men were smokers. No significant differences were seen in the frequency of smoking in men in the two age groups (≤40 y or >40 y). 66.2% of the men consumed alcohol. However the distribution of alcohol use was similar in men ≤40 y or >40 y. Varicocele was detected in 253/470 (53.8%) of men, and the frequency was not significant (55.9% and 46.6%) in men ≤40 y or >40 y. The incidence of grade 2 varicocele was higher than grade 1 (49.6% vs. 39.6%) although this was comparable between men ≤40 y (49.3%) or >40 y (51.1%). The distribution of the semen parameters in these groups is shown in Table 2. Among the alcohol users, the antioxidant concentration was lower in the 31 - 40 y group compared to the ≤30 y (p <0.024).

**Table 2:** **Comparison of semen parameters between overall and 4 age groups among varicocele, smokers and alcohol users**

Group	Parameter	Overall	≤30 y	31 – 40 y	<40 y	>40 y
		(n = 472)	(n = 69)	(n = 298)	(n = 367)	(n = 105)
Varicocele (n = 253)	Volume (mL)	3.1 ± 1.5	3.4 ± 1.5	3.1 ± 1.5	3.2 ± 1.5	3.1 ± 1.9
	Concentration (X10^6^/mL)	42.01 ± 50.58	33.43 ± 39.19	31.25 ± 37.10	31.67 ± 37.42	35.91 ± 44.58
	Motility (%)	44.7 ± 19.7	46.3 ± 14.5	41.9 ± 19.9	42.5 ± 19.1	40.0 ± 22.1
	Normal Morphology (%)	3.2 ± 2.9	3.1 ± 2.6	2.8 ± 2.8	2.9 ± 2.7	2.9 ± 2.8
	TAC (micromolar trolox)	1964.42 ± 683.77	1985.10 ± 674.43	1842.23 ± 699.77	1985.10 ± 674.43	1977.51 ± 737.31
	ROS (RLU/sec/X10^6^)	267.7 (59.3; 1277.3)	1078.7 (37.7; 8611.2)	484.7 (94; 2144.3)	316.6 (90.5; 1197.7)	265.6 ± 234.8 (41; 1322.7)
	Sperm DNA damage (%)	19.9 ± 15.3	18.9 ± 11.0	17.6 ± 11.1	17.7 ± 11.7	22.0 ± 15.9
Smokers (n = 100)	Volume (mL)	3.1 ± 1.5	2.9 ± 1.4	3.0 ± 1.4	3.0 ± 1.4	2.6 ± 1.4
	Concentration (X10^6^/mL)	42.01 ± 50.58	30.86 ± 33.13	44.20 ± 50.23	42.14 ± 48.07	53.89 ± 77.41
	Motility (%)	44.7 ± 19.7	45.2 ± 16.2	43.9 ± 17.5	44.2 ± 17.2	49.9 ± 25.5
	Normal Morphology (%)	3.2 ± 2.9	3.8 ± 2.8	3.1 ± 3.0	3.2 ± 3.0	3.5 ± 2.6
	TAC (micromolar trolox)	1964.42 ± 683.77	2146.67 ± 503.39	1842.23 ± 699.77	1883.75 ± 681.28	1683.08 ± 873.09
	ROS (RLU/sec/X10^6^)	267.7 (59.3; 1277.3)	1078.7 (37.7; 8611.2)	484.7 (94; 2144.3)	485 (90.6; 2238.3)	743.2 (215.8; 2744.2)
	Sperm DNA damage (%)	19.9 ± 15.3	18.9 ± 11.0	17.6 ± 11.1	17.8 ± 11.0	16.0 ± 10.9
Alcohol users (n = 296)	Volume (mL)	3.1 ± 1.5	3.1 ± 1.5	3.1 ± 1.5	3.1 ± 1.3	3.1 ± 1.7
	Concentration (X10^6^/mL)	42.01 ± 50.58	33.43 ± 39.19	31.25 ± 37.10	43.67 ± 51.69	41.07 ± 55.82
	Motility (%)	44.7 ± 19.7	46.3 ± 14.5	41.9 ± 19.9	44.7 ± 20.2	45.3 ± 25.5
	Normal Morphology (%)	3.2 ± 2.9	3.1 ± 2.6	2.8 ± 2.8	3.2 ± 2.9	3.2 ± 3.1
	TAC (micromolar trolox)	1964.42 ± 683.77	2250.50 ± 513.30	1898.15 ± 672.16^a^	1944.48 ± 660.19	1940.45 ± 733.36
	ROS (RLU/sec/X10^6^)	267.7 (59.3; 1277.3)	390.6 (150.5; 2434.8)	244.4 (59.3; 866.8)	265.6 (66.2; 964.6)	346.9 (58.2; 1494.3)
	Sperm DNA damage (%)	19.9 ± 15.3	16.6 ± 12.3	19.8 ± 16.2	17.7 ± 11.7	24.1 ± 19.1

The results are presented as mean ± SD for all the parameters except ROS which is presented as median (25^th^; 75^th^ percentile).

^a^ *P* value <0.05 when 31-40 y group was compared with ≤30 y group.

## Discussion

The effect of paternal age on semen quality is controversial. To help clarify the issue, we looked at a number of variables relating to semen quality in men older and younger than 40 y of age. We first looked at conventional semen parameters and found that they were not associated with advancing age, which is comparable to results of previous studies [14, 18, 48]. These men were not typically ‘old’ and hence we did not see dramatic change in the semen parameters compared to the younger group.

The lack of differences in semen parameters between the age groups seen in our study may be attributed to the patient enrollment. These patients are likely to have significant spermatozoa damage. This does not rule out the possibility that these same parameters may worsen with age should this study be done among the general population. Furthermore, this was a retrospective study, however a longitudinal study examining the effect of age on sperm parameters especially DNA damage would be ideal especially in a non-selected population. All patients attending our center were in the reproductive age group interested in initiating a pregnancy. We reported the overall percentage motility only and did not classify it into different categories.

We next looked at paternal age and oxidative stress levels. Oxidative stress, the state of imbalance between production of ROS and antioxidant capacity, is known to affect male fertility potential [49]. Although the effect of ROS on male fertility has been extensively investigated, there is lack of data on the relationship between advanced paternal age and seminal ROS levels. Cocuzza et al. evaluated semen samples of 98 fertile and healthy men and found a correlation between advanced male age (over 40 y) and high levels of ROS [22]. In this study, we did not find a significant association between advanced male age and seminal ROS levels. Similarly, TAC levels were not significantly different in males over 40 y old compared to that of males below 40 y of age.

Sperm DNA damage was assessed in the present study as well. It has been associated with low fertilization rates, increased risk of abortion and increased incidence of disease in offspring [50]. It is also considered a strong predictor of male fertility [51]. In our study, we found an association between advancing male age and sperm DNA damage using the TUNEL assay. This finding is consistent with numerous other studies that have shown increases in sperm DNA damage with advancing paternal age using different sperm DNA damage measurement techniques including the sperm chromatin structure assay (SCSA) [52, 53], single-cell gel electrophoresis (COMET) [54], and TUNEL assay [41]. Contrary to this, some studies did not find any significance of advancing male age on sperm DNA damage [21, 23, 24]. Plastira et al. showed increase in sperm DNA damage with age only in infertile patients with oligoasthenoteratozoospermia (OAT) but there was no difference in the control group [25].

In the current study, we found that infertile men >40 y were at higher risk of sperm DNA damage compared to younger men. Studies which reported correlation between male age and sperm DNA damage in infertile subjects also showed significant increase in sperm DNA damage in infertile men over 40 y [41, 53]. Moskovtsev et al. reported doubling of sperm DNA fragmentation in men ≥45 y compared to men <30 y [42]. This negative impact of paternal age on sperm DNA damage can occur earlier in infertile men, as reported in a study that included 508 infertile men and showed a significant increase in sperm DNA damage in men ≥35 y [43]. However, the risk increased significantly after the age of 40 y. Another study included 61 infertile men with oligoasthenoteratozoospermia (OAT) that were divided into two groups (≤34 y and ≥35 y). These investigators reported significant increase in sperm DNA fragmentation in patients who were ≥35 y compared to the younger group [25].

Several studies have shown a decline in fertility and reproductive outcomes in men over 40y [55]. In a large European multicenter study which included more than 3000 couples from 4 countries, the risk of infertility significantly increased if the male partner of a woman aged 35-39 y was >40 y [33]. The same data also showed increase in the risk of miscarriages in couples where the woman was ≥35 y and the man was ≥40 y [10]. In another retrospective study, Hellstrom et al. [56] tested semen parameters in 1174 men aged 45 y and older to derive the semen and sperm reference ranges by age quartiles and compare to the established WHO 1999 reference values [57]. However, unlike our study group, which was limited to age of the men seeking infertility help, their study comprised of 4 groups of healthy subjects (general population) without infertility problems. Their age ranged from >45 y (group 1); >47.8 to 51.5 y (group 2); >51.5 to 56.6 y (group 3) and >56.6 to 80.1 y (group 4). In their study the mean age was 52.9 y, only 46% of the study subjects actually met or exceeded the WHO reference values [57]. Their goal was to derive the age matched reference values for semen parameters.

In addition they also stratified their population based on age, smoking history, alcohol consumption or serum hormone concentrations. One of our study limitations was that it was a retrospective study. Another limitation was that we did not examine the clinical diagnosis of our infertile population and the patients were grouped based only on the age. In addition, the data obtained in the current study features a select population, i.e. that of infertile men attending a tertiary care hospital. Another reason for lack of significant differences in semen parameters may be because of the patient selection in different age groups. Ideally, investigation of the effect of age on sperm quality and the relevant DNA damage should be carried out using a non-selected population, i.e. investigating different age groups enrolled independently of any infertility problems. This approach will allow the assessment of age as an independent risk factor and allow for confirmation that semen parameters worsen with age. However, this is a challenge as on-going semen samples from healthy individuals are not easily available.

The impact of advanced paternal age on ART outcomes is still controversial [48]. Studies examining this relationship are lacking, except the limited data supports that paternal age >40 y is associated with failure to conceive with IVF and ICSI. One such study is by De La Rochebrochard et al. who investigated the effect of paternal age on IVF outcomes. They examined 59 IVF centers from France with a total of 1938 men whose partners were totally sterile (bilateral tubal absence or obstruction). The authors reported high risk of failure to conceive after conventional IVF when the fathers were >40 y [58]. The possible explanation for such negative influence of advanced paternal age on fertilization and reproduction was the sperm DNA damage. An intact sperm DNA is essential for fertilization and for the genetic transmission [23, 36]. Sperm DNA fragmentation is associated with several adverse outcomes including reduced fertility [37], increased miscarriage rates [38], abnormal embryonic development [39], and compromised chromosomal integrity in the embryo [40]. Li and co-workers reported in their meta-analysis that high degree of sperm DNA damage, as assessed by TUNEL assay, significantly reduces the chances of clinical pregnancy through IVF, but not ICSI. However, sperm DNA damage when assessed by SCSA showed no significant effects on clinical pregnancy rates after IVF and ICSI. Furthermore, sperm DNA damage (assessed by TUNEL assay) did not significantly affect fertilization rates in IVF and ICSI [59].

In the present study, we found than men over 40 y are at high risk of sperm DNA damage, and this may explain the findings of previous reports which showed decline in fertility, increased miscarriages, increased chromosomal and genetic disorders, increased risk of low birth weight, and decreased success of assisted reproduction. Duration of infertility in our study was higher in men >40 y compared to those ≤30 y.

Although the incidence of varicocele in the general population is about 15%, majority of the infertility patients attending our male infertility clinic present with a clinical varicocele. In our study, the overall incidence of varicocele was 53.8% (253/470); and the distribution was not very different in the 3 age groups i.e. 56.5% (39/69) in <30 y; 38.9% (116/298) in 31 - 40 y and 46.6% (48/103) in >40 y group. We did not examine other clinical causes of male infertility besides the incidence of primary and secondary infertility or diagnosis of varicocele. We also studied the incidence of smoking and alcohol in these patients. Finally, we did not find a significant association between smoking, alcohol use or incidence and grade of varicocele in men ≤40 y with those >40 y.

The clinical practice guideline of the Society of Obstetricians and Gynecologists of Canada (SOGC) recommends to counsel partners about the potential risk of seeking pregnancy when the male partner is over 40 y [60]. The American College of Obstetricians and Gynecologists (ACOG) also recommends counseling the partners with advanced male age (≥40 y) although they do not recommend any screening tests but to treat the pregnant partner as one would with any other pregnancy [29].

There is a general consensus that paternal ageing is associated with decline in semen parameters and increasing number of sperm with DNA damage. A recent review highlights the effects of advanced paternal ageing on semen parameters as well as IUI outcome [61, 62]. In the study by Beloc et al. sperm DNA fragmentation was related to poor motility in patients with select sperm defects i.e. isolated oligozoospermia, isolated asthenozoospermia and isolated teratozoospermia. In our study we grouped patients based on the age only, and it is likely that this may have contributed to the significant overlap in the values of semen parameters and lack of significant differences [63]. We did not examine the correlation of sperm DNA damage with IVF outcome. It would be interesting to see the correlation between sperm DNA fragmentation and IVF outcome in the ART population; however it must be noted that the population of our retrospective study were infertile males presenting for semen analysis and not ART candidates. Mitochondria are powerhouse organelle. They play an important role in ATP synthesis, cell signaling, reactive oxygen species by oxidative stress production as well as apoptosis. The decline in germ cells, embryo development as well as in implantation failure and miscarriage is related to mitochondrial–dependent apoptosis [64, 65]. The germ cell genome decay is the major cause of male infertility and these findings emphasize the need for molecular tools to examine the genetic and epigenetic changes affected in male infertility.

Findings of our study imply the potential negative impact of age on sperm DNA damage. We suggest that couples should be counseled about the potential risks of delayed parenthood when the female partner is >35 y and the male partner is >40 y. Supplementation with some exogenous antioxidants such as melatonin could be considered to help reduce apoptosis and improve DNA integrity in these patients [66–69].

In conclusion, advanced paternal age (>40 y) in infertile men increases the risk of sperm DNA damage. Evaluating sperm DNA damage in men of this age group is important as it may compromise their fertilization ability and increase the risk of poor ART outcome, and several chromosomal and genetic disorders. Future studies are required to investigate the effects of compounding factors such as varicocele, smoking or alcohol use on the effect of ageing on sperm DNA damage.
